# Integrating Intestinal Ultrasound in the Personalized Management of IBD

**DOI:** 10.3390/jpm16040199

**Published:** 2026-04-01

**Authors:** Cristina Lanzotti, Mariangela Allocca, Alessandra Zilli, Ferdinando D’Amico, Virginia Solitano, Sara Massironi, Silvio Danese, Federica Furfaro

**Affiliations:** 1 Gastroenterology and Gastrointestinal Endoscopy Division, IRCCS San Raffaele Hospital, Via Olgettina 60, 20132 Milan, Italy; lanzotti.cristina@hsr.it (C.L.); allocca.mariangela@hsr.it (M.A.); zilli.alessandra@hsr.it (A.Z.); damico.ferdinando@hsr.it (F.D.); solitano.virginia1@hsr.it (V.S.); massironi.sara1@hsr.it (S.M.); danese.silvio@hsr.it (S.D.); 2 Faculty of Medicine and Surgery, Vita-Salute San Raffaele University, Via Olgettina 56, 20132 Milan, Italy; 3 Gastroenterology Unit, Istituti Ospedalieri Bergamaschi, 24046 Bergamo, Italy

**Keywords:** intestinal ultrasound, IBD, ulcerative colitis, Crohn’s disease

## Abstract

Personalized medicine is increasingly shaping the management of inflammatory bowel disease (IBD), with the goal of tailoring diagnostic and therapeutic strategies to individual patients. Intestinal ultrasound (IUS) has emerged as a pivotal, non-invasive, and repeatable tool for assessing disease activity, treatment response, and complications in both Crohn’s disease and ulcerative colitis. Beyond its role in routine monitoring, IUS enables real-time decision-making and facilitates tight control strategies, aligning with the principles of precision medicine. By combining morphological assessment with advanced techniques, such as contrast-enhanced ultrasound and elastography, IUS offers unique opportunities for risk stratification and individualized treatment planning. Moreover, its accessibility, safety, and patient acceptability make IUS particularly suited for longitudinal follow-up and early detection of therapeutic failure, thereby reducing the need for invasive procedures. This review discusses the integration of IUS into personalized IBD care pathways, highlighting current evidence, clinical applications, and future perspectives.

## 1. Introduction

### 1.1. Evolution of Personalized Medicine in IBD

Inflammatory bowel disease (IBD) consists of two main subtypes, Crohn’s disease (CD) and ulcerative colitis (UC), plus a third, IBD-U, which is unclassified. Each subtype has distinct features, but is characterized by chronic, relapsing, and remitting intestinal inflammation, resulting in various complications and a reduced quality of life [1]. Although there is still no definitive cure for IBD, a broad range of treatment options exists to manage disease flare-ups and extend periods of remission for patients with mild to severe forms, available through various routes of administration. Historically, first-line therapies such as corticosteroids, mesalazine, and immunosuppressants—including thiopurine, methotrexate, and cyclosporine—have yielded mixed results, often causing side effects or failing to adequately control moderate-to-severe cases, thereby driving the development and clinical adoption of parenteral biologic therapies with targeted anti-inflammatory mechanisms, as well as oral small-molecule drugs, offering new hope for improved disease management [2]. Personalizing treatment and selecting the most appropriate therapy for each patient are essential, as it is already practiced in oncology and immunotherapy [1], and applying this personalized approach to IBD management offers potential for improving results and reducing exposure to therapies that are ineffective or potentially harmful [2]; consequently, more focus has been placed on establishing new time-specific treatment targets and modifying medications in IBD management within a treat-to-target approach, supported by algorithms to assist clinicians in their decisions, and, simultaneously, treatment effectiveness should be tracked using non-invasive methods, aiming to obtain clinical and histological remission, enhance quality of life, and avoid disability [3].

These goals could be achieved via a top-down method, where monoclonal antibody therapy is used as the initial treatment to alter the natural course of the disease [4], or through a more conservative strategy: in moderate-to-severe CD, it helps induce clinical remission, while a step-up treatment approach may be appropriate for milder cases, minimizing unnecessary risks for the patient [5]. Before starting biologic therapy, we consider certain patient characteristics, such as exposure to microbial agents, to limit toxicity and drug-related complications and to avoid reactivation of latent infections [6], and, similarly, before starting an immunosuppressant like thiopurines, analyzing the gene panel related to their metabolism can help lower the risk of myelosuppression or liver failure [7]. In patients receiving anti-TNF-alpha therapy, monitoring drug levels and testing for antibodies against the medication can provide valuable insight into whether the treatment requires adjustment, enabling assessment of true loss of response, which would necessitate a change in treatment approach [8]. The PANTS study examined anti-TNF-alpha treatments in CD and found a strong link between the HLA-DQA1*05 allele and higher levels of anti-TNF antibodies, and testing for this HLA haplotype can help clinicians tailor treatment decisions, potentially recommending immunomodulators to reduce risk and improve outcomes [9].

### 1.2. Role of Non-Invasive Monitoring Tools

As IBD is a chronic condition, it necessitates regular outpatient monitoring every 3 to 6 months, with more frequent evaluations if symptoms worsen to modify treatment plans, highlighting the need for non-invasive, patient-friendly methods that minimize discomfort, improve adherence, and support follow-up care, and, ideally, such methods should include quick, affordable, and highly accurate tests [10,11]. Clinical monitoring of IBD relies on various scoring systems developed and refined over time to assess disease activity. One of the key tools used in CD, the Crohn’s Disease Activity Index (CDAI score), was introduced in 1976 and includes eight variables [12], but over the years it has been refined, leading to the creation of a shortened version, which closely correlates with the full index, still remaining a valid, reliable, and responsive tool for assessing disease activity [13]. In 1980, the Harvey–Bradshaw Index (HBI) was developed as a further simplification of the CDAI [14], offering an intuitive approach to clinical practice that requires no laboratory markers [15]. In monitoring UC, the Mayo Score, established in 1987, is the most commonly used tool for assessing disease activity, evaluating the condition based on three main factors: the frequency of bowel movements, the occurrence of rectal bleeding, and endoscopic findings, alongside to the Physician’s Global Assessment [16,17].

Laboratory markers have long been used to differentiate IBD from other conditions, assess disease activity, predict relapses, evaluate the risk of complications, and monitor treatment response [18]; however, they cannot pinpoint the exact location or extent of the disease [18]. C-reactive protein (CRP) is a widely used, cost-effective marker with a short half-life [19], making it responsive to changes in inflammation [20] and, although it correlates moderately with endoscopic disease activity, it remains a non-specific marker, as it may rise in other systemic inflammatory conditions and remain normal even in the presence of inflammation [21]. This highlights the need to develop specific markers for assessing intestinal activity, such as fecal calprotectin (FC), a protein from neutrophils that is highly sensitive to intestinal inflammation [22] and reflects the movement of granulocytes through the intestinal wall, providing a straightforward and affordable method for monitoring IBD, detecting subclinical inflammation early and allowing for intervention before clinical symptoms escalate [23,24].

While colonoscopy remains the gold standard for diagnosing and monitoring IBD, a newer, less invasive method is gaining popularity for outpatient follow-up: intestinal bowel ultrasound (IUS) [25]. This non-invasive imaging technique provides real-time, cross-sectional images of the intestines, enabling clinicians to assess disease activity and treatment response in IBD patients and to make informed decisions about patient management during routine visits [26,27]. Combining these non-invasive tools (FC+IUS)—each with its own sensitivity and specificity—could improve disease monitoring while reducing the need for invasive procedures [28].

### 1.3. Growing Evidence

Endoscopic examination remains the gold standard for assessing disease activity, screening, and surveillance through biopsies to detect inflammation-related dysplasia [22], being crucial for evaluating mucosal healing, a key treatment goal outlined in the STRIDE II recommendations [3]. However, this procedure is invasive, often requiring sedation or anesthesia, and is associated with high cost [18] and significant environmental impact, the latter including factors such as patient and staff transportation, the use of disposable materials, staff travel, and the energy consumption needed for procedure rooms, including heating and electricity [29]. Moreover, cross-sectional imaging, such as CT and MRI, like endoscopy, has a higher environmental impact compared to ultrasound, due to energy consumption, the use of contrast agents, and the need for specialized equipment and facilities, contributing to a larger carbon footprint [30]. Endoscopy has certain diagnostic limitations, as it mainly focuses on the mucosal layer and does not provide insights into transmural involvement or extra-intestinal complications, such as enlarged lymph nodes, small amounts of ascites, inflammatory mesenteric fat (iFAT or fat wrapping), loss of wall stratification, or disease localized near the ileum [31,32,33].

IUS can effectively address endoscopy’s diagnostic limitations by pinpointing intestinal strictures and detailing their features, such as the length of the affected segment, diameter, and upstream dilation [34], resorting to contrast agents like Sonovue or hexafluoride can help distinguish between perfused and non-perfused tissues, aiding in the detection of conditions such as phlegmons, abscesses, fistulas, inflammatory pseudotumors, adhesions, or inflamed small bowel loops [35]; by providing such detailed imaging, IUS enables more precise treatment planning and helps distinguish between patients who require surgery and those who can be managed non-surgically, thereby optimizing patient care [36]. IUS can serve as a useful supplementary tool for distinguishing between UC and CD, although confirmation through endoscopy and histological examination is still necessary [37], and it provides prognostic information regarding disease progression, as the normalization of wall thickness is strongly associated with clinical response after 12 weeks of treatment, reinforcing its importance in evaluating treatment results and supporting clinical decisions [38,39]. The advantage of using IUS in outpatient practice lies in its ability to assess disease activity and treatment response in real time, providing insights comparable to those of endoscopy and MRI, but in a more accessible and less invasive manner [40].

## 2. Core Principles and Methodological Framework of IUS in IBD

### 2.1. Technical Aspects

**Figure 1:**
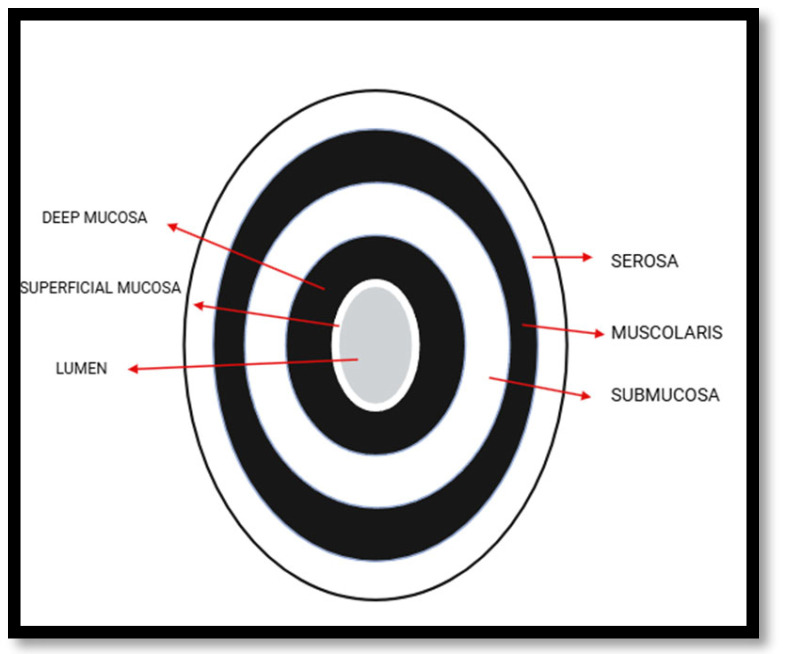
Visualization of bowel layers in IUS. Lumen: echogenic or anechoic, depending on its contents; superficial mucosa: hyperechoic; deep mucosa: hypoechoic; submucosa: hyperechoic; muscolaris: hypoechoic; serosa: hyperechoic.

**Figure 2:**
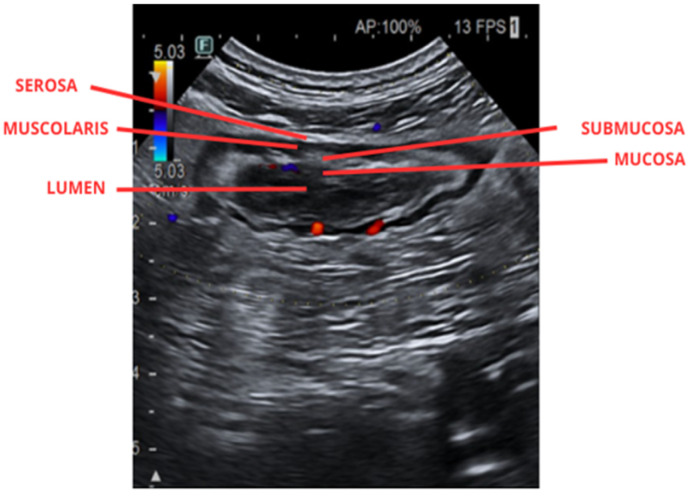
Ultrasound image of the intestine illustrating bowel wall thickening and the stratified appearance of the bowel wall layers.

The exam is carried out using two probes with different frequencies: a low-frequency convex probe, which provides greater ultrasound depth to study the abdomen globally and detect potential complications, followed by a higher-frequency probe to obtain a more detailed, high-resolution image of the five layers of the intestinal wall loops, each with different echogenicity (Figure 1 and Figure 2) [41].

Another type of probe that can be used is the high-frequency (5–13 MHz) microconvex probe, capable of providing high-quality, high-resolution images [41]. These layers, extending from the innermost lumen to the serosa, are visualized to enable measurement of each segment of the bowel wall thickness (BWT), an ultrasound parameter used to evaluate disease activity and treatment response, being less than 3 mm in a condition of remission, with measurements taken perpendicular to the wall in both longitudinal and transverse planes [32]. Vascularization is another key parameter for assessing disease activity, measurable through color Doppler, with velocities usually ranging from 5–7 cm/sec, while alternative imaging methods like power Doppler, advanced dynamic flow, or Superb Microvascular Imaging (SMI), are less influenced by angle variations and can offer further details about the disease condition [32].

The examination is performed systematically, usually starting in the left iliac fossa, where the sigmoid colon is located, using landmarks such as the left iliac artery, vein, and psoas muscle for orientation, and applying gradual compression to reduce gas interference and improve visualization of the wall layers, then the scan progresses proximally through the colon, reaching the splenic flexure and thick-walled transverse colon near the stomach, then moves toward the right iliac fossa, using similar reference points to assess the ileocecal region [27,41]. The small intestine is then examined using the “lawnmower technique,” in which the probe is moved in overlapping parallel strips to cover the full area of the loops [27,41]. IUS can also be performed via a transperineal approach, using a probe covered with an examination glove or sterile sheath to evaluate and treat perianal fistulas and the rectal walls, which may be less clear with transabdominal IUS, while being less invasive than endoanal ultrasound [42,43]. The rectum, compared to other intestinal segments, is more challenging to examine, along with the jejunum and the first 30 cm of the ileum [27], because it lies deep within the pelvis, far from the abdominal wall, and its visualization can be hindered by intestinal gas [44]. Transperineal ultrasound (TPUS) can be used both to assess perianal involvement in CD and to evaluate rectal inflammation in patients with UC, and, in this regard, Sagami et al. observed that TPUS strongly correlates with both endoscopic severity and histologic activity, providing a detailed assessment of the disease’s severity and extent when paired with a transabdominal approach [44].

### 2.2. IUS Parameters and Score

BWT, measured as the perpendicular distance from the luminal surface to the serosa, is a key parameter evaluated during ultrasound, and it should be assessed in each segment examined, with remission indicated by a cutoff value of less than 3 mm for the small intestine and less than 4 mm for the colon in adults [45] and measured at the level of the anterior bowel wall in the longitudinal direction, avoiding the inclusion of haustrations and mucosal folds [46]. Another important factor evaluated during the examination using color Doppler is vascularization, classified into four grades based on the semiquantitative Limberg score, determined by the number of vessels identified per square centimeter: Grade 0 corresponds to a normal appearance, with BWT within normal limits, preserved bowel wall stratification (BWS) and absence of wall vascularization, grade 1 is defined by a thickened bowel wall without vascular signals, grade 2 by the presence of short stretches of vascularity, grade 3 by the presence of longer stretches of vascularity and grade 4 by vascular signals extending into the surrounding mesentery [47]. Together with an increased wall thickness, it is indicative of active disease. Other features that may indicate active disease include fibro-fatty proliferation, particularly in patients with chronic CD, which appears as hyperechoic tissue surrounding the affected intestinal loops [48], as well as abnormalities not directly linked to disease activity, such as regional lymphadenopathy, the presence of free fluid [48,49], a localized change in BWS or an alteration spanning more than 3 cm of an inflamed segment [50] (Table 1).

Over the years, various scoring systems have been developed that combine the key parameters mentioned above to simplify the assessment of disease status in outpatient settings, help differentiate between active and inactive disease, and predict long-term patient outcomes. IBUS-SAS, is calculated using the formula 4 × BWT (mm) + 15 × intestinal fat + 7 × CDS + 4 × BWS and is used in CD to assess the presence of segmental disease activity [51], with 23.8 as an optimal cut-off for diagnosing activity and 50 for moderate-to-severe activity [52] and so is BUSS-Bowel ultrasound score (0.75 × BWT + 1.65 × CDS; where CDS = 1 if present, or CDS = 0 if absent), with values indicative of remission <3.52 and an ultrasound response defined with a score decrease of 1.2 [53]. In UC, MUC (Milan Ultrasound Criteria = 1.4 × BWT (mm) + 2 × BWF; where BWF = 1 if present, or BWF = 0 if absent) is indicative of a state of remission if ≤6.2 [38] (Table 2).

IUS also provides a fast and accessible way to evaluate complications such as areas of fibrosis, commonly seen in CD, which appear as hyperechoic, spiculated bands extending from the submucosa toward the mesentery [34], while strictures are identified by thickened bowel walls, often with a significantly reduced or absent lumen, lacking peristalsis, and occasionally accompanied by proximal bowel dilation [56,57]. Fistulas can be identified by the presence of hypoechoic segments, which may or may not contain hyperechoic material, located between bowel loops or between other structures that are abnormally in contact, such as the bladder, skin, or mesentery [58] and intestinal abscesses present as hypoechoic areas containing fluid and gas artifacts, with posterior enhancement and indistinct borders, and are avascular, while ulcers appear as localized depressions within the mucosal layer [48].

By using practical parameters and scoring systems, IUS allows clinicians to efficiently evaluate disease progression and treatment effectiveness, without relying on more invasive or less accessible diagnostic methods.

### 2.3. Feasibility

IUS is becoming an increasingly popular examination in clinical practice, primarily due to its feasibility, as it doesn’t require fasting, unlike colonoscopy or radiological methods with contrast agents, which are necessary for optimal results [42] and, although European Federation of Societies for Ultrasound in Medicine and Biology (EFSUMB) guidelines recommend fasting for at least 4 h, this does not substantially enhance visibility, but it facilitates the assessment of small bowel motility and the vascularization of the splanchnic vessels [59]. Additionally, IUS removes the need for laxatives or bowel preparation, except when performing small intestinal contrast ultrasonography (SICUS), which can be uncomfortable for patients and occasionally result in incomplete examinations [60]. Ultrasound follow-up offers significant advantages for pregnant patients, as it is radiation-free and generally better tolerated than endoscopic procedures and, despite some limitations in visualization due to the gravid uterus [61], it remains a useful tool even in later stages of pregnancy, although third-trimester scans may have reduced visibility of the small bowel. As highlighted by De Voogd et al., IUS activity showed a moderate-to-strong correlation with clinical activity and FC levels, achieving 84% sensitivity and 98% specificity for distinguishing active from quiescent disease when FC was combined with clinical activity [62]. This underscores the importance of achieving both clinical and biological remission to minimize risks for both the mother and fetus [62].

IUS is also valuable for monitoring postoperative patients for potential recurrences, as a BWT greater than 3 mm at the anastomotic site or in the terminal ileum serves as a reliable indicator of recurrence [56], thereby reducing the need for more invasive or less accessible tests such as MRI while maintaining high sensitivity and specificity. Ultrasound methods such as IUS, SICUS, and contrast-enhanced ultrasound (CEUS) demonstrate high sensitivity and specificity in detecting postsurgical recurrence in CD: a BWT over 5.5 mm is linked to severe recurrence, and although ultrasound is very effective for diagnosing postoperative recurrence, SICUS shows greater sensitivity but is slightly less specific than traditional bowel sonography [63]. Additionally, increased BWT, elevated FC, and the presence of lymph nodes have been shown to be independent predictors of post-operative recurrence, especially when combined, identifying up to 75% of patients with endoscopic recurrence with no need for colonoscopy [64].

IUS is especially valuable for less compliant patients, like children, by customizing ultrasound scoring to the specific patient group (e.g., UC-IUS, MUC, SPAUSS, and Civitelli Index), and this approach demonstrated a strong correlation between these ultrasound scores and clinical, biochemical, and endoscopic activity in pediatric UC [65]. Ultrasound score IBUS-SAS (International Bowel Ultrasound Segmental Activity Score), a CD activity score developed by Novak et al. that integrates BWT, BWS, color Doppler vascularization, and iFAT [51], was able to predict endoscopic treatment response to infliximab with high accuracy in pediatric CD patients [66].

## 3. Positioning IUS Among Imaging Modalities in IBD

### 3.1. Comparison with Other Imaging Modalities (MRI, CT, Endoscopy)

#### 3.1.1. Inflammatory Bowel Disease: Role of Endoscopy and Cross-Sectional Imaging

For many years, colonoscopy has been the gold standard for evaluating lesions and disease activity of the colon and terminal ileum in patients with IBD [39]. It allows direct visualization of the mucosa, facilitating the assessment of mucosal healing, now considered a key therapeutic goal within the treat-to-target approach [3], as well as evaluating disease extent and activity, monitoring therapeutic response, and collecting biopsies for histological analysis. However, colonoscopy is not always feasible due to its invasive nature, the need for bowel preparation, and costs. It can also yield false-negative results, especially in CD when inflammation is confined to the proximal small intestine. Additionally, colonoscopy primarily provides information about the mucosal surface, making it less suitable for evaluating transmural inflammation and extraintestinal disease [33]. Over time, cross-sectional imaging methods such as CTE (Computed tomographic enterography), MRI (Magnetic Resonance enterography), and IUS have become increasingly important for evaluating IBD, as they are less invasive than endoscopy and provide a more detailed picture of the disease. These techniques overcome some limitations of endoscopy by examining the entire gastrointestinal tract, measuring BWT, and detecting extra-intestinal complications [33].

#### 3.1.2. Crohn’s Disease

In CD, mucosal healing alone is insufficient, as disease activity often extends beyond the mucosa. The concept of transmural healing, defined as the resolution of full-thickness bowel wall inflammation, has therefore gained increasing relevance. Although no universally accepted definition exists, a 2022 consensus suggests that it would be indicated by an ultrasound finding of a BWT of less than 3 mm and the absence of vascularization on color Doppler imaging [40]. Achieving transmural healing has been associated with improved long-term outcomes, including a reduced need for surgery, and appears superior to mucosal healing alone, even after discontinuation of biologic therapy [67,68]. Nevertheless, approximately one-third of patients with endoscopic remission fail to achieve transmural healing [68]. Several studies have compared IUS with endoscopy and cross-sectional imaging techniques such as CT and MRI in CD. The METRIC trial demonstrated that both IUS and MRE are valid first-line techniques for disease assessment, with MRE showing higher sensitivity for disease activity and detection of extraintestinal complications, while IUS displayed comparable specificity [69]. A systematic review reported that IUS is an accurate technique for diagnosing suspected CD and evaluating disease activity, although its performance is inferior to MRE in disease proximal to the terminal ileum [70]. Handheld ultrasound (HHUS) has demonstrated diagnostic accuracy comparable to MRE for CD diagnosis, although MRE remains superior for defining disease extent and detecting complications, such as strictures, abscesses, and fistulas [71]. Another systematic review comparing MRE and IUS with endoscopy for monitoring drug response in CD found that reductions in BWT on IUS correlated well with endoscopic remission in 10 studies, while 12 studies showed that the MaRIA score (Magnetic Resonance Index of Activity) on MRI reliably predicts endoscopic remission [72]. A non-inferiority diagnostic study reported that, while IUS was slightly less accurate than MRI in defining CD extension and detecting enteroenteric fistulas, there was high concordance in CD location between the two methods (k = 0.81) [73]. In a comparison between MRE and IUS in combination with colonoscopy, IUS showed high accuracy in determining disease location, activity, and complications, and the concordance of therapeutic decisions based exclusively on IUS or MRE compared with clinical decision-making was similar (≈0.77), as was the agreement between IUS and MRE (0.80) [74]. Regarding the use of IUS in therapeutic decision-making, Novak et al. reported that IUS influenced therapeutic modifications in approximately 60% of cases based on ecographic findings [75]. The correlation between the main ultrasound scores and the SES-CD (Simple endoscopic score for Crohn’s Disease) and Rutgeerts scores showed a significant positive correlation [*p* < 0.0001], especially for IBUS-SAS [ρ = 0.87], also in severe clinical and endoscopic activity [76].

Videocapsule endoscopy (VCE) is another valuable tool in the assessment of small bowel in CD. Ukashi et al. found a significant correlation between the Lewis score and terminal ileum BWT (r = 0.597, *p* < 0.001), as well as with the IBUS-SAS (r = 0.647, *p* < 0.001), early in the study, which weakened as time progressed [77]. The relationship was moderate for treatment response and modest during remission, with optimal terminal ileum BWT cut-offs of 2.25 mm for mild inflammation and 3.6 mm for moderate-to-severe inflammation [77]. A meta-analysis showed that VCE has slightly higher specificity and overall diagnostic performance than IUS for detecting SB-CD [78], and performed better than IUS in assessing treatment response in ileocolonic CD, although its use may be limited by strictures [79].

CT as a diagnostic tool was more sensitive than IUS (90.7% vs. 88.4%) but less specific (83.3% vs. 93.3%), but IUS was superior in detecting stenoses (10 vs. 8 n° of patients), skip lesions (7 vs. 4) and fistulas (11 vs. 8), detecting more cases with mesenteric lymphadenopathy (26 vs. 9) and equal cases with abscesses and free peritoneal fluids [80]. Due to exposure to ionizing radiation, CTE is commonly employed when timely access to IUS or MRE is unavailable or contraindicated, in elderly patients for whom radiation exposure poses fewer concerns, and in acute situations [22] (Table 3).

#### 3.1.3. Ulcerative Colitis

The assessment of mucosal healing in UC, defined as both endoscopic and histological remission [81], remains a key goal in managing the condition, even though a universally agreed-upon definition has yet to be established. Reaching this state correlates with improved long-term results, such as fewer steroid cycles or surgeries, lower hospitalization rates, and a decreased risk of cancer [82].

IUS in UC has improved on some endoscopy limitations, allowing for highly sensitive and specific detection of colon inflammation, though it still faces challenges when examining the rectal walls [83], predicting a treatment-specific response within just a few weeks of starting therapy [84] and accurately predict whether a patient with acute severe ulcerative colitis (ASUC) will respond to steroids within 48 h, using changes in BWT to allow quick intervention if rescue therapy is needed [85]. In a case–control study, a comparison between colonoscopy and IUS in UC activity was made, showing the ability of IUS in early detection of disease flares and consequent management, potentially delaying or avoiding unnecessary colonoscopies, it can also be used for monitoring disease progression and assessing short-term treatment responses [86]. In a prospective study, MUC in UC disease activity assessment were found to be highly accurate with a high interobserver agreement, showing a significant correlation between ecographic features and endoscopic activity [*p* < 0.05] [87], and a MUC score > 6.2 has been confirmed to be a valid cut-off to distinguish active from non-active UC with a sensitivity of 0.85 specificity of 0.94, significantly correlating with Mayo endoscopic sub-score (*r* = 0.76; *p* < 0.0001) [88], also being a strong predictor of long-term endoscopic response in UC patients undergoing biologic therapy [89]. A good correlation was found between BTW reduction, Mayo endoscopic score, and Robarts Histopathologic index at baseline and after 2 months of therapy with tofacitinib, with a BWT < 2.8 mm being the most accurate cut-off for endoscopic remission, 3.9 mm for improvement, and a decrease of 32% for response [90], supporting the value of BWT change as non-invasive marker of therapeutic effect.

### 3.2. Safety, Repeatability, and Patient Acceptability

Given the chronic nature of IBD, serial follow-up is essential, and IUS is increasingly used in clinical practice due to its accessibility, low cost, non-invasiveness, and patient-friendly nature. Rajagoplan et al. through the use of questionnaires assessed the acceptability and tolerance of the examination in IBD patients, rating it on a visual analog scale from 0 to 10, found that IUS was rated as highly acceptable by patients (mean VAS 9.20/10), significantly higher than colonoscopy, stool and blood sampling, and other imaging (*p* < 0.01) [91], while in a nationwide study in CD reported that both IUS and blood sampling were significantly more acceptable than colonoscopy, mainly due to fear of complications and discomfort [92]. In direct comparisons, IUS was the most tolerated exam, followed by MRE, and colonoscopy was the least preferred, mainly due to the preparation process and the pain or discomfort experienced during the procedure [93]. FC testing can be tricky, too, as it involves handling stool samples and retrieving the necessary kit, and as previously mentioned, it is not specific for determining disease localization. In contrast, IUS can be conducted as a point-of-care (POC) procedure during the patient visit, without requiring additional time, offering real-time results, enabling immediate clinical decisions to be made [94].

Over the years, the development of ultrasound scores with specific cut-off values for ultrasound parameters has improved the reliability of IUS in determining disease activity and localization, enhancing reproducibility across different operators and centers and reducing inter-observer variability, which is mainly influenced by operator experience. A multicenter study by Fraquelli et al. emphasized the need for standardizing common ultrasound parameters, demonstrating good-to-excellent reproducibility for several key indicators [95]. For instance, BWT in CD showed k-values ranging from 0.7 to 1, indicating strong agreement, while free fluid (0.85–1) and stenosis (0.81–1) exhibited excellent concordance, and bowel wall fibrosis (BWF) also showed good k-values (0.53–0.89), whereas bowel wall pattern (−0.22–0.85) and mesenteric hypertrophy (0.14–0.69) had poorer values [95]. Calabrese et al. demonstrated that interobserver variability for the same parameters among six operators from different international centers was significantly minimized, likely due to the implementation of more standardized protocols and pre-defined ultrasound parameters [31]. To address the absence of a reproducible activity index, Novak et al. developed the IBUS-SAS scoring system, which evaluates four key parameters of segmental inflammation in CD, BWT, BWS, hyperemia, and iFAT, demonstrating excellent reliability, with an intra-class correlation coefficient (ICC) of 0.97 (*p* < 0.001) [51]. Using expert consensus and standardized techniques, essential indicators of disease activity on IUS were identified, yielding a segmental activity score with high reliability [51].

Compared to other commonly used methods for monitoring IBD patients, IUS is one of the safest options, providing radiation protection, as it does not use ionizing radiation like CTE, and avoiding the risks associated with endoscopic procedures and, furthermore, for patients with pacemakers, there is no concern about MRI compatibility. As previously mentioned, IUS is also safe for pregnant women, though some limitations in visibility may arise due to the physiological changes of pregnancy, allowing for the assessment of disease activity and treatment response without the risks associated with endoscopy, which could potentially affect the placenta and fetus [96,97].

## 4. Personalized IBD Management Through IUS-Guided Strategies 

### 4.1. IUS-Guided Treatment Optimization (Tight Control, Treat-to-Target)

As new evidence and therapies emerge, there’s a growing need to identify therapeutic targets beyond just symptoms, which may not always reflect the true inflammatory process, since residual disease activity—both biochemical and endoscopic—can be observed in 50% of patients in clinical remission [98]. Research has shown that a Treat-to-Target (T2T) approach enhances long-term outcomes by emphasizing early interventions and rigorous disease management, particularly in CD [99]. Initially used in the management of chronic conditions, T2T was integrated into IBD care following the release of the (International Organization For the Study of Inflammatory Bowel Disease (IOIBD) STRIDE guidelines in 2015, which set clear therapeutic goals, emphasized ongoing monitoring and treatment adjustments until these objectives were met, and prioritized clinical remission and mucosal healing as the main targets [100]. Nevertheless, emerging evidence pointed to the need for an update, leading to the release of the STRIDE II guidelines in 2021, which introduced time-based targets for reaching therapeutic goals and expanded the focus to include factors such as prognosis, quality of life, and patient-reported outcomes, underscoring the importance of controlling inflammation through regular monitoring with biomarkers and imaging [3]. Among the secondary objectives set by STRIDE II are mucosal healing in CD and histological healing in UC, which may offer additional benefits beyond mucosal healing alone; however, current evidence remains limited in supporting these targets, partly because available therapies are ineffective in achieving them [100]. STRIDE-II continues to recognize IUS as a transformative tool for disease monitoring, given its ability to evaluate activity along the entire intestine and to determine whether transmural healing has occurred [3]. The introduction of time-based targets has significantly advanced the concept of “tight control,” a strategy that emphasizes managing inflammation through consistent, frequent patient monitoring, aiming to prevent long-term complications by adjusting treatment based on biochemical markers, even when symptoms are absent. As a result, it has led to improved clinical and endoscopic outcomes, including achieving steroid-free remission [101]. This approach was assessed in the CALM trial for managing patients with early CD using two distinct strategies, demonstrating that therapy escalation with adalimumab, guided by both symptom changes and biomarker levels, led to better clinical and endoscopic outcomes than adjustments based solely on symptoms [102], and it was also shown to be cost-effective compared to traditional clinical management, as reported by an economic analysis [103].

Regarding the application of IUS in the management and optimization of IBD treatment, Saleh and Abraham were the first to report its potential as a point-of-care tool. This method allows for the gathering of objective data on disease activity, which complements biochemical markers, helping to tailor treatment and resulting in notable improvements in the disease condition for a substantial number of patients during follow-up [104], as also reported by data from Vaughan et al., indicating that POCUS (Point-of-Care Ultrasound) impacts treatment decisions in 60% of cases, facilitating therapy optimization, modification, or de-escalation [105].

IUS is an ideal tool for a tight control strategy due to its non-invasive nature, repeatability, and cost-efficiency, as it enables continuous monitoring of disease activity and treatment response across different stages—early, intermediate, and late—allowing for prompt adjustments to therapy.

### 4.2. Early Response Assessment and Prediction of Outcomes

Numerous studies demonstrate that IUS is a highly reliable method for predicting IBD outcomes, showing a strong correlation between IUS scores and endoscopic results, especially concerning long-term remission and improvement, and it effectively predicts critical factors such as disease activity, complications, and treatment response. A correlation between BTW measurement and endoscopic response was found by de Voogd et al. in patients with moderate-to-severe UC at weeks 2 and 6 after starting treatment, showing that a 3.52 mm BTW could predict mucosal healing with high sensitivity and specificity, and a BWT of 2.98 mm had 100% specificity for identifying endoscopic response [84], also revealing that response times varied depending on the therapy, reporting an early BTW reduction for infliximab and tofacitinib, visible by week 2, while the effects of vedolizumab became noticeable only after 6 weeks of treatment [84]. CD patients who achieved transmural healing after 2 years of anti-TNF-alpha therapy demonstrated significantly better outcomes, including higher rates of steroid-free remission, fewer hospitalizations, and no surgeries, compared with those with mucosal healing or no healing, contributing to longer time to relapse [67]. In a multicenter study by Calabrese et al., transmural healing in CD varied by therapy, with the infliximab group achieving the highest rates; additionally, BWT demonstrated significant improvement from baseline, with higher baseline values associated with a lower likelihood of achieving transmural healing at subsequent assessments [106]. An elevated IBUS-SAS score at diagnosis was found by Madsen et al. to be correlated with later need for biologic therapy, surgery, or hospitalization, with patients with a score above 80 all requiring hospitalization and initiation of systemic steroids [107]. In UC, a MUC score greater than 6.2 may indicate the need for therapy escalation, steroid use, or more aggressive disease progression; conversely, patients with a baseline MUC score below 6.2 had a significantly lower likelihood of these outcomes [54].

IUS enables early evaluation of treatment response after the start of new therapies or before altering the treatment plan, underscoring the value of using ultrasound early to distinguish responders from non-responders, enabling prompt adjustments and stopping ineffective treatments. In the TRUST&UC Trial, Maaser et al. demonstrated that IUS can detect a therapeutic response in UC patients within just days or weeks after treatment begins, often faster than in CD patients, showing that BWT normalized in approximately half of the UC patients within two weeks, being strongly correlated with clinical improvement [39]. Significant improvements in all ultrasound parameters at 3 and 12 months following treatment intensification were also shown in CD by Kucharzik et al., and an association between BTW and CRP reduction was observed at 3 months [108], while a correlation between BTW reduction, endoscopic Mayo score, UC Endoscopic Index for Severity and Robarts Histopathologic Index was observed after 8 week of tofacitinib in UC [90]. A systematic review of 28 studies examined the predictive capacity of IUS for treatment response across multiple time points in CD, UC, and ASUC, showing that ultrasound response was evident in the early phases of therapy [109].

### 4.3. IUS in Assessing Mucosal Healing and Transmural Healing

While there is no universally accepted definition, mucosal healing has traditionally been synonymous with endoscopic healing, which refers to the resolution of inflammation-related lesions and damage that can be directly visualized during an endoscopic procedure [110], and, more recently, definitions of mucosal healing have started to incorporate histologic healing; however, this concept remains poorly defined and lacks agreement among clinicians and experts [111]. Because mucosal healing has prognostic value for disease outcomes, non-invasive methods are crucial for long-term monitoring. Parente et al. investigated the role of IUS in patients with severe UC receiving high-dose corticosteroids as an alternative to colonoscopy and found strong and consistent agreement between the 0 and I Baron scores and ultrasound measurements, with a moderate-to-severe Baron score associated with a high risk of endoscopic activity at 15 months [112], while Yzet et al. found that a BWT < 3 mm predicted endoscopic mucosal healing with 56% sensitivity, 88% specificity, 95% PPV, and 36% NPV, and when a BWT < 3 mm was combined with absent color Doppler vascularization and FC < 250 µg/g, all these predictive parameters showed improvement [113].

Although not yet officially recognized as a target in the STRIDE-II guidelines, transmural healing in CD, defined as the resolution of mucosal ulcerations, transmural inflammation, and extramural disease, remains an important therapeutic goal [114], as it is associated with favorable long-term outcomes, as observed in the CALM study, showing a correlation between deep remission, involving both endoscopic and clinical ones, plus free-steroid remission, and transmural healing, with a lower risk of disease progression [102], but also in the previously reported study by Castiglione et al. [67]. Geyl et al. conducted a systematic review of studies on transmural healing in CD, evaluating both clinical outcomes and biomarkers, and exploring various imaging techniques such as MRI and IUS, reporting that transmural healing assessed via IUS was typically defined as a BWT of less than 3 mm, absence of vascularization on CDS, preserved BWS, and no signs of complications, such as lymph node enlargement or mesenteric fat hypertrophy [115]. The review also highlighted long-term outcomes, showing a strong correlation between transmural healing and favorable results, including reduced rates of surgery and hospitalization, as well as higher rates of steroid-free remission [115]. While the findings are encouraging, a standardized definition of transmural healing has yet to be established, and there is no consensus on the most reliable markers and imaging techniques for evaluation. Transmural healing should be regarded as a crucial treatment objective in CD, alongside traditional goals such as mucosal healing, as it may improve disease management, reduce complications, and improve long-term outcomes, including fewer hospitalizations, lower surgical rates, and more sustained remission.

## 5. Challenges and Strategies for Reliable IUS Practice 

### 5.1. Operator Dependence (Is It True)?

A common limitation of IUS is that its accuracy depends on the operator’s skill, as diagnostic precision relies on the operator’s experience. However, this challenge also applies to all diagnostic techniques that involve professional interpretation of images [42]. Some researchers have sought to evaluate the extent to which the operator’s skills affect image interpretation and explore the learning curve involved in mastering this technique. Bezzio et al. investigated how IUS accuracy improved with the operator’s experience and whether prior abdominal ultrasound experience facilitated learning, finding a steady increase in agreement as the operator’s experience grew, with more experienced trainees reaching proficiency more quickly. To align with the expert, trainees had to complete a minimum of 84 exams to recognize increased BWT and 79 exams to detect bowel dilation, while detecting intra-abdominal complications, considered an advanced skill, required at least 97 exams [116]. Bove et al. showed that an experienced gastroenterologist achieved strong agreement with an expert radiologist in IUS after 50 exams, with no clear learning curve, whereas a novice showed poor agreement overall, improving more rapidly only in patients without prior IBD surgery [117]. Van Wassenaer et al., presenting data from the pediatric field, found that non-radiologist physicians could be trained to perform IUS with diagnostic accuracy comparable to radiologists, although inter-observer agreement in assessing disease activity was only moderate (k 0.49–0.58) [118]. Aside from operator dependence, patient-related factors can also interfere with the interpretation of ultrasound images, including high body fat, thin bowel walls, a tortuous bowel course, or excessive gas [119,120].

Properly trained physicians can perform IUS with accuracy comparable to experts, with sufficient inter-observer agreement for IBD management, and the main limitations are technical rather than interpretive, underscoring the need for thorough training and extensive hands-on experience [116,117].

### 5.2. Standardization and Reproducibility

The lack of standardized protocols and reproducibility limits the clinical application of IUS, further compounded by the absence of a global consensus on standardizing techniques, interpretation, performance, and training guidelines for its use [41,42]. A significant variability in ultrasound-based definitions of response and remission was reported in a systematic review by Allocca et al., underscoring the importance of standardizing suitability criteria and outcome measures for the application of IUS in clinical trials [121]. Ilvemark et al. in 2022 conducted a systematic review and expert consensus to define ultrasound targets, monitoring timelines, and assessment criteria for remission and transmural healing, but variability in response criteria and evaluation timing across studies limited comparability with treatment outcomes [40]. In a 2025 expert consensus, Allocca et al. sought to establish standardized ultrasound criteria for assessing response and remission in IBD, highlighting the need for technical consistency and operator expertise to ensure reproducibility in multicenter trials, outlining key parameters for evaluation, including BWT, CDS, BWS, iFAT, submucosal thickening, and disease extent [122]. In luminal CD and UC, an ultrasound response is defined by a ≥25% reduction in BWT or by combined improvement in BWT and other IUS parameters, while ultrasound remission involves normalization of BWT and/or multiple parameters, though in UC, the normal sigmoid BWT range is uncertain, and a key point raised was that UC might be classified as a transmural disease because of submucosal thickening [122]. The consensus also recommends specific assessment timings, with more time required to evaluate the ileum. Only MUC and BUSS scores were approved for use in clinical trials, whereas no consensus was reached on IBUS-SAS due to inconsistent cutoff values across studies [122].

IUS shows promise as a tool for evaluating treatment response in IBD. However, its use is limited by the lack of standardized protocols, definitions, and monitoring timelines. Although reviews and expert consensus have made progress, additional standardization is required to enhance consistency and reliability and to encourage broader clinical adoption.

### 5.3. Economic and Logistical Considerations

Multiple studies have assessed the accuracy, sensitivity, and specificity of the primary cross-sectional imaging methods used to evaluate IBD, with IUS demonstrating similar accuracy to CTE and MRE, especially in detecting localized disease activity, stenosis, or abscesses [70,74]. While CT and MRI provide superior resolution and detailed assessment of disease complications [123], IUS is a valuable alternative for long-term monitoring of IBD patients because it is radiation-free, allowing for frequent checks, and is also more cost-effective. An economic sub-analysis of the METRIC trial showed that IUS is much more cost-effective than MRE and can reduce the average waiting time, highlighting its potential to enable faster treatment for patients with CD [124]. A study conducted at a tertiary center in Southeast Asia looked into the economic impact of using IUS instead of endoscopic exams or MRE, showing a significant cost saving and highlighting the environmental benefits of IUS, with a substantial reduction of CO_2_ emissions [125], while in a similar study conducted at a tertiary center in Singapore, IUS was found to eliminate the need for MRE in nearly 80% of cases and reduce the reliance on colonoscopies by 58.8%, leading to notable cost saving and lower CO_2_ emissions, further emphasizing the dual advantages of IUS in both cost and environmental sustainability [126]. Moreover, nurse-led triage and the implementation of rapid-access intestinal ultrasound (RAIUS) to manage IBD flare-ups could significantly reduce unplanned hospital admissions and urgent clinic visits, leading to substantial savings in hospital costs [127].

A major reason IUS is seen as a cost-effective choice is its logistical ease, as it can be performed at the point of care during outpatient visits, eliminating the need for fasting and simplifying the process for patients while speeding up diagnosis and facilitating faster clinical decisions [42,75]. POCUS provides a practical alternative to more invasive and it has demonstrated excellent diagnostic accuracy. When evaluated against cross-sectional imaging, it showed a sensitivity of 87.2% and a specificity of 87.5%, establishing itself as a dependable and efficient tool for managing these patients [128]. High sensitivity and specificity were also observed in detecting disease activity, although values were somewhat lower when evaluating disease extent [129]. POCUS can play a crucial role in clinical decision-making, especially when it comes to detecting early signs of relapse in patients with CD, and, by providing rapid, on-site results, it helps reduce the need for further tests, shortens the waiting times for diagnostic outcomes, and allows for more timely management of patients, ultimately improving care efficiency [130].

The availability of lightweight, portable, and compact HHUS devices has promoted the widespread use of this practice, now extending beyond outpatient settings to include home visits and operations in remote or underserved regions [131]. HHUS has proven to be a cost-effective, reliable, and accurate tool for assessing IBD, especially in situations where traditional larger machines like cart-based ultrasound (CBUS) are not feasible, providing perfect specificity (100%) for detecting active disease and aligning well with other imaging techniques, even in patients with higher BMI. Few discrepancies have been reported; therefore, in such instances, it is advisable to use POCUS in conjunction with other disease activity markers or confirm results with CBUS for enhanced accuracy [132]. HHUS and conventional IUS exhibited comparable accuracy in assessing disease extent in UC, with strong agreement in evaluating disease activity, as reflected by the MUC score (k = 0.86, *p* < 0.01), indicating high consistency between the methods [133]. POCUS is a non-invasive, real-time tool for assessing IBD that improves decision-making and outcomes while reducing the need for more expensive and invasive tests, making it a valuable addition to current management strategies.

## 6. Innovations and Future Perspectives in Intestinal Ultrasound

### 6.1. Artificial Intelligence in IUS

Artificial intelligence is increasingly shaping medicine by using machine learning and deep learning systems to integrate large datasets, support earlier diagnosis, improve prognosis and treatment planning, and enhance follow-up, all within the growing framework of personalized medicine [134,135]. Machine learning enables a system to analyze data, recognize patterns, and improve its performance through repeated training, allowing it to apply learned models to new data for descriptive and predictive tasks [136,137]. Deep learning, a branch of machine learning, aims to mimic the brain’s ability to analyze data such as images, sounds, and text by using multilayer neural networks to help machines automatically identify complex patterns, eliminating the need for manual feature engineering [138,139]. Different types of neural networks are designed for particular tasks: Convolutional Neural Networks (CNNs) are especially good at processing spatial and visual information, whereas Recurrent Neural Networks (RNNs) are well-suited for sequential data, enabling them to detect patterns and produce precise predictions [140].

The implementation of AI within gastroenterology, particularly in the realm of endoscopy, is a significant milestone in the early detection of gastrointestinal tract preneoplastic and neoplastic lesions through the enhancement of the adenoma detection rate and the development of new scoring systems for disease-risk stratification, prognostic assessment, and better monitoring of therapeutic response [138]. In IBD as well, where the complex and multifactorial nature of the condition requires a tailored approach to patient care, AI is employed not only in the endoscopic setting but also in cross-sectional imaging techniques. Liu et al. assessed the diagnostic performance of machine learning models using radiomic features extracted from MRI images of the terminal ileum and clinical data to predict small bowel-Crohn’s disease (SB-CD) in pediatric patients, with support vector machine models trained on radiomic features from manually segmented regions of interest and clinical variables. Their models, especially those combining T2-weighted radiomics with clinical variables, achieved higher diagnostic accuracy than expert radiologists and reduced interobserver variability [141]. Wasnik et al. demonstrate that machine learning techniques can automate the detection and spatial localization of key qualitative features in CD on CTE images, such as mural enhancement, stratification, stenosis, wall thickening, and mesenteric fat stranding, with accuracy comparable to that of experienced radiologists [142].

Mucosal healing, primarily assessed endoscopically, is a key target for improving long-term outcomes; Ma et al. created a CNN that utilized baseline IUS images combined with clinical data to forecast mucosal healing in CD after one year of treatment. The model demonstrated moderate accuracy and primarily concentrated on features of the bowel wall, serosal surface, and nearby mesentery [143]. Integrating AI into ultrasound practice could serve as a valuable solution to improve reliability and reproducibility by automating the detection of key inflammatory markers and disease complications [144]. Carter et al. developed and validated an AI-driven system using CNNs to distinguish between bowel wall thickening and normal bowel images in IUS for CD patients, demonstrating impressive performance, achieving an accuracy of 90.1%, sensitivity of 86.4%, and specificity of 94% in detecting bowel wall thickening, with an AUC of 0.9777 [145], while in the pediatric field, Kumaralingam et al. developed an AI-based algorithm designed to automatically measure BWT identifying the interface between the layers of the bowel wallwith a sensitivity of 90.29% and specificity of 93.70% when using a 2 mm BWT threshold, also demonstrating strong agreement with expert evaluations, with an intraclass correlation coefficient (ICC) of 0.942, highlighting its reliability [146]. Gu et al. explored the potential of radiomic analysis to differentiate between disease activity and normal conditions in IUS images, comparing the performance of a radiomic-based model with a CNN-based classification model. The radiomic model using XGBoost performed very well (AUC 0.98, ~94% sensitivity/specificity, ~94% accuracy), whereas the CNN model showed lower performance (AUC 0.75) [147].

### 6.2. CEUS, SICUS and Elastography

Quantitative IUS techniques provide objective numerical measurements of intestinal tissue using specialized software or advanced ultrasound modes, improving image quality and the accuracy of key parameters, and helping overcome the limitations of standard IUS in clinical practice. Vascularization is typically assessed by Color Doppler analysis, with intensity measured according to the semi-quantitative Limberg score [47]. CEUS advances ultrasound imaging by using injected microbubble contrast to objectively quantify inflammatory activity in real time (such as Sonovue, typically administered at a dose of 2.4–4.8 mL with saline), that quickly perfuses areas with active intestinal disease. Inflammatory activity is quantified using specialized software integrated into the ultrasound device, which analyzes the enhancement pattern, peak intensity, and washout time. Additionally, dynamic contrast-enhanced ultrasound (DCE-US) further tracks intensity changes over time to assess progression of inflammation [148,149]. The EFSUMB guidelines highlight CEUS as a highly valuable tool, not only for assessing disease activity in patients with CD but also for monitoring complications like abscesses and fistulas. Furthermore, CEUS helps differentiate between inflammatory lesions, which show strong contrast enhancement, and fibrotic lesions, which are less responsive to contrast agents [150]. In a prospective study by de Voogd et al., CEUS effectively differentiated inflammatory lesions from chronic strictures in post-resection patients, and the newly proposed Stricture Score Amsterdam demonstrated excellent accuracy and strong interobserver agreement [151].

SICUS is an additional method that improves the visualization of the small intestine using oral contrast (nonabsorbable polyethylene glycol dissolved in water) that highlights the bowel, assessed retrogradely with the probe, enabling detection of strictures, fistulas, and the extent of the disease [152,153], showing high sensitivity and specificity (98.7% and 100%, respectively) in the detection of small bowel lesions in pediatric CD, with excellent agreement (κ = 0.84) for strictures when confronted to MRE [154] and high sensitivity in detecting strictures of 87.5% and 100% for pre-stenotic dilation in a comparison to surgery [155]. Prujit et al. conducted a systematic review and meta-analysis of B-mode IUS and SICUS for detecting strictures, inflammatory masses, and fistulas. B-mode demonstrated good sensitivity and specificity, while SICUS performed even better, with higher pooled sensitivity and specificity across all lesion types [58].

Elastography is an ultrasound technique that non-invasively distinguishes fibrotic from inflammatory lesions by measuring bowel wall stiffness through acoustic or mechanical stimulation [36], which includes three methods: a low-frequency acoustic radiation force impulse (ARFI) generated by an IUS device and shear-wave elastography (SWE), both quantitative techniques, and strain elastography (SE), a qualitative method [36,156]. Shear wave elastography creates mechanical shear waves in tissue using focused acoustic pulses, whose speed is measured by the ultrasound system and directly correlates with tissue stiffness. Fibrotic tissues allow faster wave propagation, so this method offers an objective way to quantify bowel wall stiffness and differentiate between fibrotic and inflammatory strictures [157,158]. Additionally, we can distinguish between point-SWE, which assesses shear wave velocity at a specific, designated Region of Interest, and 2-dimensional-SWE, which creates a color-coded map to visualize shear wave velocity across a wider area [36]. Strain elastography evaluates tissue deformation caused by compressive force using repeated pulses, offering a semi-quantitative measure via the strain ratio, which compares the deformation of the bowel wall to that of reference tissue, such as adjacent mesenteric fat. Additionally, we can distinguish between point-SWE, which assesses shear wave velocity at a specific, designated Region of Interest, and 2-dimensional-SWE, which creates a color-coded map to visualize shear wave velocity across a wider area [159] or qualitative assessment (stiffness expressed on a color map) [160]. In a study of 35 post-resection CD patients after surgical resection, SWE values one week post-op were higher in severe fibrosis than in moderate or mild cases, and a cut-off of 22.55 KPa distinguished severe from mild/moderate fibrosis with 69.6% sensitivity, 91.7% specificity, and an AUC of 0.82 [157]. Lu et al. found that in ileal Crohn’s disease, SWE values were higher in operated patients and correlated with smooth muscle hypertrophy rather than fibrosis. CEUS peak enhancement inversely correlated with fibrosis and SWE velocity, suggesting SWE may reflect muscle hypertrophy while CEUS helps distinguish active from chronic inflammation [161]. Real-time elastography with strain ratio measurement did not correlate with the histological fibrosis score in CD patients who underwent surgery and was unable to reliably differentiate between fibrotic and inflammatory tissue, likely due to the simultaneous presence of both inflammation and fibrosis [162], while Baumgart et al. reported that real-time elastography can be used to detect intestinal fibrosis in stricturing CD, with RTE strain values being significantly lower in fibrotic bowel segments [163].

When combined, conventional B-mode ultrasound, CEUS, and strain elastography improve the ability and accuracy to visually differentiate inflammatory from fibrotic ileal strictures in patients with CD compared with any single modality alone, with no change in inter-reader agreement [164].

### 6.3. Remote Monitoring and Tele-Ultrasound

Managing IBD requires ongoing monitoring, but frequent visits to specialized centers can be difficult for some patients, especially those in rural or remote areas. In recent years, technological advancements have significantly transformed medical diagnostics and patient care. The development of systems that enable remote acquisition, interpretation, and sharing of ultrasound images has made healthcare more accessible, streamlined, and efficient. These innovations help reduce waiting times and improve the overall responsiveness of healthcare management, ensuring that patients receive timely care regardless of their location [165,166,167]. Tele-ultrasound, a form of telemedicine, uses network-connected ultrasound devices to transmit images to remote healthcare professionals, enabling diagnosis and monitoring without the patient being physically present. During the procedure, the ultrasound is performed either by healthcare staff or non-specialized personnel, with images sent to a qualified expert for interpretation. This can be done in real-time (synchronous), where the operator and expert communicate live, or asynchronously, where images are sent for later analysis [168,169]. To illustrate the potential of remote monitoring in IBD, Krugliak Cleveland et al. shared the case of a multi-failure UC patient who was trained to use a handheld ultrasound device to monitor the thickness and vascularization of the sigmoid colon daily after starting a JAK inhibitor. Images were sent to the medical team, who reviewed and confirmed their quality. Although the patient was clinically stable, a gradual increase in BWT prompted the medical team to proceed with surgical intervention [170]. To further highlight the effectiveness of remote monitoring, Del Hoyo et al. assessed the impact of a web-based system, TECCU, for managing complex IBD in 63 patients, compared with standard care and nurse-assisted telephone care. The study found that the TECCU group had the highest remission rate at 81%, followed by standard care at 71.4%, and telephone care at 66.7%, contributing to better disease management, fewer healthcare visits, and overall improvements in both patient quality of life and satisfaction [171].

## 7. Conclusions

**Figure 3:**
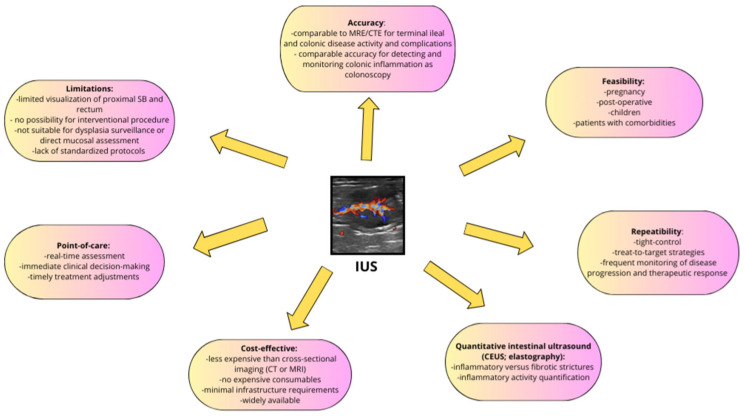
Key characteristics, advantages, and limitations of intestinal ultrasound in diagnostic and monitoring imaging and clinical practice.

IUS has emerged as an increasingly valuable tool in IBD management, providing a non-invasive, cost-effective, and real-time method for monitoring disease activity, assessing treatment response, and guiding clinical decisions. It reliably evaluates disease markers such as BWT and detects complications, including strictures and abscesses, with accuracy comparable to CT and MRI, especially in routine and POC settings where fast results are crucial. IUS also supports personalized care by providing continuous, patient-specific data that helps clinicians adjust therapy promptly, improving outcomes and reducing delays. However, wider adoption is limited by the lack of standardized protocols and the need for skilled operators, underscoring the importance of structured training. Future research should focus on standardizing imaging protocols, enhancing training, and conducting long-term studies on outcomes, cost-effectiveness, and healthcare impact. As these advances progress, IUS is expected to play an increasingly central role in comprehensive IBD care (Figure 3).

## Figures and Tables

**Table 1:** IUS parameters with corresponding pathological findings.

IUS PARAMETERS	DEFINITION	PATHOLOGICAL FINDING
Bowel wall thickness	Perpendicular measurement in mm from the luminal boundary to the serosa	Ileocolic: >3 mm Rectum: >4 mm
Color Doppler signal (CDS)	Blood Flow Assessment in the blood vessels of the intestines	Limberg Score >2
Bowel wall stratification	Distinct bowel layers seen as alternating hyperechoic and hypoechoic bands	Focal or extended loss of delineation of the layers
Mesenteric lymph nodes	Hypoechoic cortex and hyperechoic hilum, well-defined borders, <5 mm on the short axis	>5 mm on the short axis
Motility	Assessment of bowel movements	Altered or absent due to inflammation or fibrosis
Haustrations	Regular, parallel lines or folds in the colonic wall	Absent, reduced, or disorganized
Inflammatory fat (iFAT)	Hypoechoic tissue surrounding the intestines and mesentery.	Fat wrapping the bowel, with an increased echogenicity

**Table 2:** Overview of the main diagnostic scoring systems employed in the evaluation of Crohn’s disease and ulcerative colitis activity.

IUS Score	Formula	Parameters	Cut-Off for Activity
CROHN’S DISEASE AND ULCERATIVE COLITIS			
IBUS-SAS [51]	(4 × BWT) + (7 × CDS) + (4 × BWS) + (15 × iFAT).	BWT: measurement in mm CDS: absent (0), short signals (1), long signals inside bowel (2), long signals inside and outside bowel (3) BWS: normal (0), uncertain (1), focal if ≤3 cm (2); extensive if >3 cm (3) i-FAT: absent (0), uncertain (1), present (2)	From 4 (no disease activity) to 100 (worst disease activity).
CROHN’S DISEASE			
BUSS [53]	0.75 × BWT + 1.65 × CDS	BWT: measurement in mm CDS: absent (0), present (1)	>3.52
ULCERATIVE COLITIS			
MUC [54]	1.4 × BWT + 2.0 × CWF	BTW: measurement in mm CWF: absent (0), present (1)	>6.2
Civitelli Index [55]	1 point for abnormal findings in IUS parameters, 0 for normal	BWT: measurement in mm Loss of bowel wall stratification Increased vascularity Absence of haustra coli	>1: moderate to severe endoscopic inflammation (ranging from 0 to 4)

**Table 3:** Comparison of sensitivity and specificity in cross-sectional imaging in CD. SE: sensitivity. SP: specificity. SB-CD: small bowel-Crohn’s disease; *p*: *p*-value; k: Cohen’s kappa; r: Spearman’s coefficient.

MRE						
Authors	Aim of the Study	MRE Sensitivity	IUS Sensitivity	MRE Specificity	IUS Specificity	Other Results
Taylor S.A. et al. [69]	Per-patient difference in sensitivity, correct identification, and localization of SB-CD.	Disease extent: 80% Disease presence: 97%	Disease extent: 70% Disease presence: 92%	Disease extent: 95% Disease presence: 96%	Disease extent: 80% (95% CI 72–86) Disease presence: 84%	SE extent: difference of 10% (1–18; *p* = 0·027) SE presence: 5% (1–9; *p* = 0·025) SP extent: difference of 14% (1–27; *p* = 0·039) SP presence: difference 12% [0–25]; *p* = 0·054)
Panès J et al. [70]	Diagnostic accuracy of cross-sectional imaging techniques.	93%	84%	90%	92%	Both have SE and SP > 0.80 for identifying fistulas, abscesses, and stenosis, but IUS produces false-positive results for abscesses.
Rispo A. et al. [71]	Comparison of diagnostic accuracy.	91.67%	87.5%	94.59%	91.89%	No significant differences (89.41% for HHUS vs. 92.94% for MRE, *p* = NS).
Castiglione et al. [73]	Comparison of diagnostic accuracy in SB-CD.	96%	94%	94%	97%	IUS was less accurate in determining CD extent (r = 0.69), with high agreement in CD location (k = 0.81) and fair concordance for strictures (k = 0.82), abscesses (k = 0.88), and enteroenteric fistulas (k = 0.67).
**VCE**						
**Authors**	**Aim of the** **Study**	**VCE** **Sensitivity**	**IUS** **Sensitivity**	**VCE** **Specificity**	**IUS** **Specificity**	**Other Results**
Shahryar et al. [78]	Head-to-head comparison and network meta-analysis of VCE vs. imaging techniques to diagnose SB-CD.	(pooled) 89.6%	(pooled) 89.3%	(pooled) 86.2%	(pooled) 72%	Ranking analysis identified VCE (*p*-score: 0.97) as the most effective diagnostic tool for SB-CD, followed by IUS, MRE, and CTE.
Brodersen et al. [79]	IUS, MRE, PCE, and FC for determining response to medical treatment in ileocolonic CD.	87.5% (95% CI, 61.7–98.4)	80.0% (95% CI, 56.3–94.3)	86.7% (95% CI, 59.5–98.3)	77.8% (95% CI, 52.4–93.6),	Activity scores decreased in patients who achieved endoscopic response: SUS-CD 2.2 vs. 6.1 (*p* < 0.001), MaRIA from 37.1 to 29.0 (*p* = 0.05), SES-CD with PCE from 12.8 to 3.1 (*p* < 0.001), and FC from 1339.9 to 115.3 mg/kg (*p* < 0.001).
**CT**						
**Authors**	**Aim of the Study**	**CT** **Sensitivity**	**IUS** **Sensitivity**	**CT** **Specificity**	**IUS** **Specificity**	**Other Results**
Zsolt et al. [80]	IUS vs. enteroclysis, CT, and immunoscintigraphy in SB-CD	90.7%	88.4%	83.3%	93.3%	Enteroclysis proved to be the most accurate method (accuracy: 98.6%), with IUS close behind at 90.4%.

## Data Availability

No new data were created or analyzed in this study. Data sharing is not applicable to this article.

## Abbreviations

The following abbreviations are used in this manuscript:

IBD	Inflammatory bowel disease
UC	Ulcerative colitis
CD	Crohn’s disease
CDAI	Crohn’s disease Activity Index
HBI	Harvey–Bradshaw Index
CRP	C-reactive protein
FC	Fecal calprotectin
IUS	Intestinal ultrasound
iFAT	Inflammatory mesenteric fat
BWT	Bowel wall thickness
SMI	Superb Microvascular Imaging
TPUS	Transperineal ultrasound
BWS	Bowel wall stratification
MUC	Milan Ultrasound Criteria
CDS	Color Doppler signal
EFSUMB	European Federation of Societies for Ultrasound in Medicine and Biology
SICUS	Small intestine contrast ultrasonography
CEUS	Contrast-enhanced ultrasound
IBUS-SAS	International Bowel Ultrasound Segmental Activity Score
CTE	Computed tomography-enterography
MRE	Magnetic resonance-enterography
HHUS	Handheld ultrasound
SES-CD	Simple endoscopic score for Crohn’s disease
ASUC	Acute severe ulcerative colitis
POC	Point-of-care
T2T	Treat-to-target
IOIBD	International Organization for the Study of Inflammatory Bowel Disease
POCUS	Point-of-care ultrasound
BUSS	Bowel Ultrasound score
RAIUS	Rapid-access intestinal ultrasound
CBUS	Cart-based ultrasound
CNNs	Convolutional Neural Networks
RNNs	Recurrent Neural Networks
SB-CD	Small-bowel Crohn’s disease
DCE-US	Dynamic contrast-enhanced ultrasound
ARFI	Acoustic radiation force impulse
SWE	Shear wave elastography
SE	Strain elastography
